# Potential Use of Tea Tree Oil as a Disinfectant Agent against Coronaviruses: A Combined Experimental and Simulation Study

**DOI:** 10.3390/molecules27123786

**Published:** 2022-06-12

**Authors:** Alice Romeo, Federico Iacovelli, Carolina Scagnolari, Mirko Scordio, Federica Frasca, Roberta Condò, Serena Ammendola, Roberta Gaziano, Maurizio Anselmi, Maurizio Divizia, Mattia Falconi

**Affiliations:** 1 Department of Biology, University of Tor Vergata, 00133 Rome, Italy; alice.romeo@uniroma2.it (A.R.); federico.iacovelli@uniroma2.it (F.I.); serena.ammendola@uniroma2.it (S.A.); 2 Laboratory of Virology, Department of Molecular Medicine, Sapienza University of Rome, 00185 Rome, Italy; carolina.scagnolari@uniroma1.it (C.S.); mirko.scordio@uniroma1.it (M.S.); federica.frasca@uniroma1.it (F.F.); 3 Istituto Pasteur Italia, 00161 Rome, Italy; 4 Department of Clinical Sciences and Translations Medicine, University of Tor Vergata, 00133 Rome, Italy; roberta.condo@uniroma2.it; 5 Department of Experimental Medicine, University of Tor Vergata, 00133 Rome, Italy; roberta.gaziano@uniroma2.it; 6 Department of Biomedicine and Prevention, University of Tor Vergata, 00133 Rome, Italy; maurizio.anselmi@uniroma2.it (M.A.); divizia@uniroma2.it (M.D.)

**Keywords:** SARS-CoV-2, coronavirus, tea tree oil, GaMD, disinfectant agent, spike glycoprotein

## Abstract

The COVID-19 pandemic has highlighted the relevance of proper disinfection procedures and renewed interest in developing novel disinfectant materials as a preventive strategy to limit SARS-CoV-2 contamination. Given its widely known antibacterial, antifungal, and antiviral properties, *Melaleuca alternifolia* essential oil, also named Tea tree oil (TTO), is recognized as a potential effective and safe natural disinfectant agent. In particular, the proposed antiviral activity of TTO involves the inhibition of viral entry and fusion, interfering with the structural dynamics of the membrane and with the protein envelope components. In this study, for the first time, we demonstrated the virucidal effects of TTO against the feline coronavirus (FCoVII) and the human coronavirus OC43 (HCoV-OC43), both used as surrogate models for SARS-CoV-2. Then, to atomistically uncover the possible effects exerted by TTO compounds on the outer surface of the SARS-CoV-2 virion, we performed Gaussian accelerated Molecular Dynamics simulations of a SARS-CoV-2 envelope portion, including a complete model of the Spike glycoprotein in the absence or presence of the three main TTO compounds (terpinen-4-ol, γ-terpinene, and 1,8-cineole). The obtained results allowed us to hypothesize the mechanism of action of TTO and its possible use as an anti-coronavirus disinfectant agent.

## 1. Introduction

The severe acute respiratory syndrome coronavirus 2 (SARS-CoV-2), first isolated in Wuhan (China) in December 2019, is a single-stranded RNA virus belonging to the Coronaviridae family, genus Betacoronavirus, and is the pathogen responsible for the ongoing COVID-19 pandemic [1]. The SARS-CoV-2 virion is composed of an outer membrane envelope, mainly coated with glycoproteins called Spike (S), which are responsible for receptor binding and virus fusion to the host cell [2]. The S protein is a trimeric class I fusion glycoprotein, highly conserved among all human coronaviruses, and is composed of an extracellular N-terminal domain (NTD), a transmembrane (TM) domain anchored to the viral membrane, and a short intracellular C-terminal domain (CTD) [3]. The NTD is fundamental for the viral entry process and is divided into two functional subunits by protease cleavage. The S1 subunit (residues 1–685) is involved in receptor recognition through its receptor-binding domain (RBD) [1,4], while the S2 subunit (residues 686–1273) mediates viral fusion and is composed of two trimeric α-helical regions called heptad repeats 1 (HR1) and 2 (HR2) [5]. After cell receptor binding, the activation of S by protease cleavage triggers huge conformational rearrangements in the HRs regions that assemble into the so-called six-helical bundle (6-HB) structure [2]. This event marks the transition of the protein from a metastable prefusion conformation to an elongated post-fusion conformation and allows upstream fusion peptides to insert within the cell membrane, providing the energy requirements needed to drive viral and cell membrane fusion and to enable viral entry into the cell [6,7]. Once entered, SARS-CoV-2 hijacks the cellular machineries to ensure its own replication and propagation [2].

Thanks to the release of effective vaccines against SARS-CoV-2, the immunization of the worldwide population has been underway since the end of December 2020 and it has been estimated that, at the time of writing this paper, more than 60% of the world population has received at least one dose of a COVID-19 vaccine [8]. However, while vaccines are highly effective in reducing patients’ hospitalization and death, they do not entirely abrogate the risk of virus infection and transmission in the vaccinated and still unvaccinated population [9].

SARS-CoV-2 can be transmitted by air or through contact with a contaminated surface [10]. One of the first studies on aerosol and surface stability showed that virus particles could be detectable for up to 3 h in aerosols, up to 24 h on cardboard surfaces, and up to 2–3 days on stainless steel and plastic [11]. Compliance with effective disinfection procedures is critical to help minimize the exposure to the virus. Since the beginning of the pandemic, the use of alcohol-based hand sanitizers containing 80% *v*/*v* ethanol or 75% *v*/*v* isopropanol was recommended by the World Health Organization [12] and the Food and Drug Administration [13] to reduce SARS-CoV-2 diffusion and decrease the healthcare burden. However, health and environmental concerns have been associated with their frequent use [14,15]. Considering the growth in the disinfectants market that is expected in the following years, considerable research efforts are being directed towards the development of innovative active substances exhibiting broad-spectrum virucidal activity, combined with low human toxicity. In particular, due to their known antiviral properties, there is considerable interest in the use of natural products as ecological and non-toxic alternatives [16,17].

Tea tree oil (TTO) is a volatile essential oil mainly obtained from the steam distillation of the leaves or branches of *Melaleuca alternifolia*, a small tree belonging to the Myrtaceae family which is endemic to Australia [18]. This oil was originally used by the aboriginal people of Australia as an antiseptic and herbal medicine, although its medicinal properties were not reported until the 1920s [19,20,21]. Since then, several studies have outlined its broad-spectrum antimicrobial activities [18,22,23,24,25,26,27,28,29], and the product is nowadays incorporated as an active ingredient in many topical formulations used for dermatological applications or oral hygiene [18,30,31,32]. Many studies also support its anti-inflammatory potential, both in vitro and in vivo, and its use as an antitumoral agent has also been suggested, since it can inhibit the growth of human melanoma cells in vitro by inducing caspase-dependent programmed cell death [18,33].

TTO is composed of volatile aromatic terpene hydrocarbons, resulting in about 100 molecular components with different concentrations [34]. Its antimicrobial properties are generally attributed to the capability of its hydrocarbon components to partition into biological membranes, impairing their structural and functional integrity [35,36,37]. Several studies have described its efficacy against enveloped viruses. In particular, TTO can reduce herpes simplex virus types 1 and 2 (HSV-1 and HSV-2) plaque formation, particularly when applied to the free virus prior to the infection of the cells [28,38], and it can also inhibit influenza virus replication in vitro [25,27,29,39]. Moreover, TTO was also proven to be highly effective in inactivating the influenza virus in vapor form, as a filter coating material or aerosolized in ambient air, suggesting its possible use as a disinfectant in bioaerosol filtration applications or in indoor air quality improvement [27,29].

**Figure 1:**
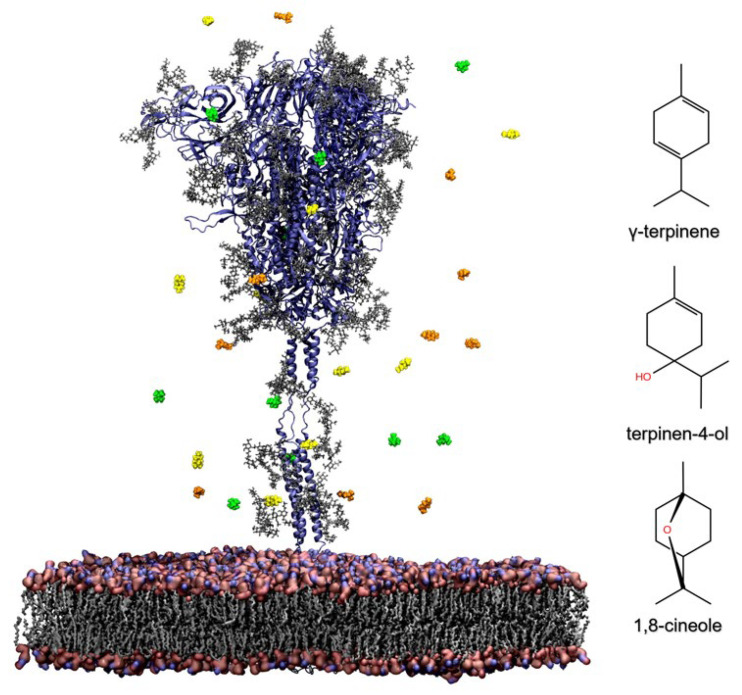
Overview of the simulation system. The S glycoprotein is shown as blue cartoon and the glycans as grey sticks. Membrane polar heads are shown as surface, colored by atom types, while lipid tails are represented as grey sticks. TTO compounds are shown as spheres, with γ-terpinene molecules in orange, terpinen-4-ol in yellow, and 1,8-cineole in green, and their molecular structure is represented in 2D on the right.

In this paper, we explored for the first time the potential use of TTO as a disinfectant agent against coronaviruses. Firstly, the in vitro virucidal activity of TTO and three of its major components (terpinen-4-ol, γ-terpinene and 1,8-cineole) was assessed using the feline coronavirus (FCoVII) and the human coronavirus OC43 (HCoV-OC43), an Alpha- and Betacoronavirus, respectively. Then, using an in silico approach, the possible activity of TTO against SARS-CoV-2 was evaluated and the experimental results obtained have been correlated to the simulations. In fact, coronaviruses share an overall similar structural organization, although structural variations between individual proteins can be observed and some viruses also display different specific accessory proteins (i.e., the HCoV-OC43 envelope includes a hemagglutinin-esterase protein not present in the SARS-CoV-2 genome [3,40]). Nevertheless, the S protein structure is overall very conserved among coronaviruses [3,41,42,43]. On the basis of this structural similarity, we performed Gaussian accelerated Molecular Dynamics (GaMD) simulations [44] of a complete model of the SARS-CoV-2 S inserted into a viral membrane portion. To investigate possible inhibition mechanisms occurring when TTO compounds diffuse over the SARS-CoV-2 outer surface, simulations were performed both in the absence or presence of the three major TTO components (Figure 1 and Table S1).

## 2. Results

### 2.1. Virucidal Activity of Tested Compounds on FCoVII and HCoV-OC43

A fixed amount of FCoVII (6 Log TCID_50_/mL) and HCoV-OC43 (5 Log RNA copies/mL) was incubated with scalar dilutions (A-C) of the TTO-EtOH formulation for 5, 15, and 30 min (Table 1). Due to the cytotoxicity of the product in CRFK and Vero E6 cells, the limit of detection of the assay was 4.50–3.50 Log TCID_50_/mL for FCoVII and 5.13 and 3.28 Log number of RNA copies/mL for HCoV-OC43 (Table 1). Results indicated that the TTO-EtOH formulation A has a strong virucidal activity against FCoVII replication in CRFK cells. In particular, at least a 3.50 Log reduction of FCoVII titer (TCID_50_/mL) was achieved after 5 min of exposure to a combination of 3.33% TTO and 5.33% EtOH (formulation A). The same results were observed after 15 and 30 min of contact of FCoVII with formulation A (Table 1). By contrast, at lower concentrations (formulations B/C), moderate or no virucidal effects against FCoVII were recorded. Having observed that TTO has potent in vitro virucidal activity against FCoVII, we also assessed the virucidal properties of these preparations against a low human pathogenic coronavirus, HCoV-OC43. As shown in Table 1, the reduction of HCoV-OC43 titer was obtained in a dose and time-dependent manner. A reduction of 1.38 Log of HCoV-OC43 RNA copies/mL was achieved after 30 min of contact with the TTO-EtOH formulation A, and a moderate anti HCoV-OC43 virucidal activity was still recorded with the other two scalar dilutions (formulations B/C). Then, the virucidal activity against HCoV-OC43 decreased after 15 or 5 min, according to the dilution used. Overall, TTO exhibited an inhibition effect on HCoV-OC43 at the concentration of 3.33%, whereas it had no significant effects starting from the concentration of 0.66% (Table 1). An examination of the virucidal activity of EtOH at 5.33% (formulation D) indicated no inhibitory effects on in vitro replication of both FCoVII and HCoV-OC43 (Table 1).

Finally, TTO virucidal activity was compared to that of ISACLEAN^®^, a commonly used disinfectant agent composed of isazone, benzalkonium chloride 50%, chlorhexidine gluconate, and isopropanol (Table 1). Already after 5 min of contact, this disinfectant induces a ≥99% virus inactivation for both coronaviruses, comparable to the effects observed for the TTO formulation A against FCoVII after 5 min, while a similar reduction was observed for HCoV-OC43 after 30 min (Table 1).

### 2.2. Virucidal Activity of Individual Components of TTO on FCoVII and HCoV-OC43

The virucidal activity of the individual components of TTO was evaluated against FCoVII and HCoV-OC43 (Table S2). Three of the major components of TTO, i.e., γ-terpinene, 1,8-cineole, and terpinen-4-ol, were selected for evaluation. As in the previously performed assays, 100 µL of each virus was incubated with 100 µL of scalar dilutions of each selected TTO component mixed with EtOH for a defined contact time of 5, 15, and 30 min (Table S2). Only the two dilutions for which a significant TTO activity was recorded were evaluated for individual compounds (3.33% and 0.66%).

The results showed that at the highest concentration tested (3.33%), all three compounds showed a high time-dependent virucidal activity against FCoVII and HCoV-OC43 (Table S2). In particular, the titer of FCoVII and HCoV-OC43 decreased by 1.00 Log (90%) and 0.89 Log (87.11%), respectively, after 15 min of contact with γ-terpinene at 3.33%. After 30 min of exposure, a ≥1.00 Log reduction, accounting for ≥90% of virus inactivation, was observed for both coronaviruses exposed to γ-terpinene. However, at a γ-terpinene concentration of 0.66%, a ≥90% reduction in viral titer was induced only for HCoV-OC43 after 30 min of contact, while the effect of the compound on FCoVII was lower.

Concerning 1,8-cineole, after 30 min of contact the compound reduced FCoVII titer by 96.84% (1.50 Log) at a concentration of 3.33%, and by 90% (1.00 Log) at a concentration of 0.66% (Table S2). On the other hand, a >90% decrease in HCoV-OC43 titer was observed with 1,8-cineole at 3.33%, already after 5 min of contact, but at 0.66% the compound is less effective, and virus titer is reduced only by 0.84 Log (85.54%) after 30 min of contact.

Interestingly, terpinen-4-ol had the strongest virucidal effects against both FCoVII and HCoV-OC43, at the highest percentage used of 3.33%, and at any time point tested (5, 15, and 30 min), causing a ≥2.15 Log (≥99.00%) reduction of the virus titers after just 5 min of contact (Table S2). However, similar to what was observed for 1,8-cineole and γ-terpinene, at a concentration of 0.66%, terpinen-4-ol showed a reduced activity against both coronaviruses, with a 90% reduction of FCoVII titer and only a 77.09% reduction of HCoV-OC43 titer achieved after 30 min of contact.

### 2.3. In Silico Evaluation of TTO Antiviral Activity on SARS-CoV-2

Considering the promising results obtained for the two coronaviruses, we hypothesized that TTO could also exert a strong virucidal activity against SARS-CoV-2. To validate this hypothesis and to atomistically elucidate the inhibitory effect carried out by TTO molecules on the SARS-CoV-2 envelope, we performed 150.0 ns of GaMD simulations [44] of a complete model of the SARS-CoV-2 S glycoprotein, including the glycosylation and palmitoylation sites, inserted in a membrane mimicking the viral envelope [45]. To account for the effect of TTO, several molecules of the three individual TTO components experimentally tested (γ-terpinene, 1,8-cineole, and terpinen-4-ol), have been randomly placed in the solvent around the protein-membrane system. The starting simulation system is shown in Figure 1. A simulation without the TTO molecules has also been performed as a comparison.

#### 2.3.1. Influence of TTO on the Viral Envelope

**Figure 2:**
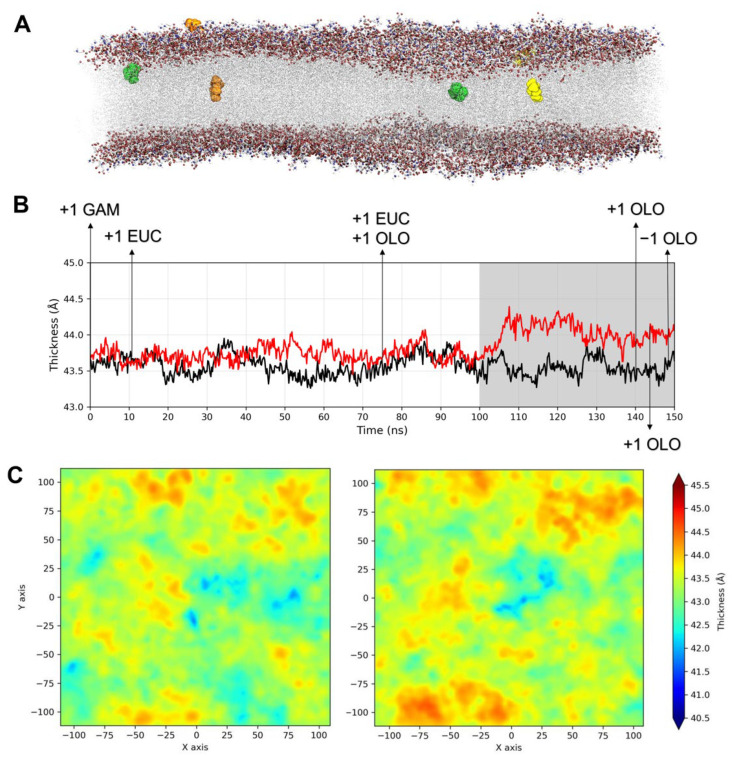
(**A**) Binding locations observed for the γ-terpinene (orange), terpinen-4-ol (yellow), and 1,8-cineole (green) molecules, shown as spheres, within the viral membrane bilayer; (**B**) membrane thickness as a function of the simulation time in the absence (black line) or presence (red line) of the TTO compounds. Arrows indicate the time-points at which the entry (+1) or exit (−1) of the molecules was observed, and the grey background highlights the thickness shift; (**C**) thickness heatmaps for the viral membrane in the absence (**left**) or presence (**right**) of the TTO molecules.

During the simulation time, six compounds, including two 1,8-cineole, one γ-terpinene, and three terpinen-4-ol molecules, entered the SARS-CoV-2 envelope, determining a clear change in membrane thickness fluctuations (Figure 2A,B). In particular, during the second half of the GaMD production phase (from about 100.0 ns until the end of the simulation), a marked increase of about 0.7 Å in membrane thickness was observed in the system with TTO (Figure 2B). At this timepoint, one γ-terpinene, two 1,8-cineole and one terpinen-4-ol molecules were inserted in membrane. The observed shift corresponds to the γ-terpinene molecule reaching the opposite membrane leaflet and the 1,8-cineole and terpinen-4-ol molecules inserting deeper into the membrane. In particular, γ-terpinene is the only compound reaching the other leaflet and also showing lateral movements, suggesting that its structure and highly hydrophobic character allow it to freely move within the lipid bilayer. Following these events, the thickness difference was stably maintained, and the other two terpinen-4-ol molecules entered the membrane, although one of them exits in the membrane a few ns before the end of the simulation (Figure 2B). Accordingly, 2D thickness maps, describing membranes thickness profiles along the z axis, outline an altered thickness pattern in the presence of TTO molecules, with the viral membrane characterized by wider regions of higher thickness (Figure 2C).

Despite the limited size of the simulation system, which was adopted to stem the computational costs, these results clearly indicate that TTO inhibition mechanism involves an alteration of the physical properties and structural organization of the viral envelope.

#### 2.3.2. Structural Analysis of TTO Interactions with the S Glycoprotein

**Figure 3:**
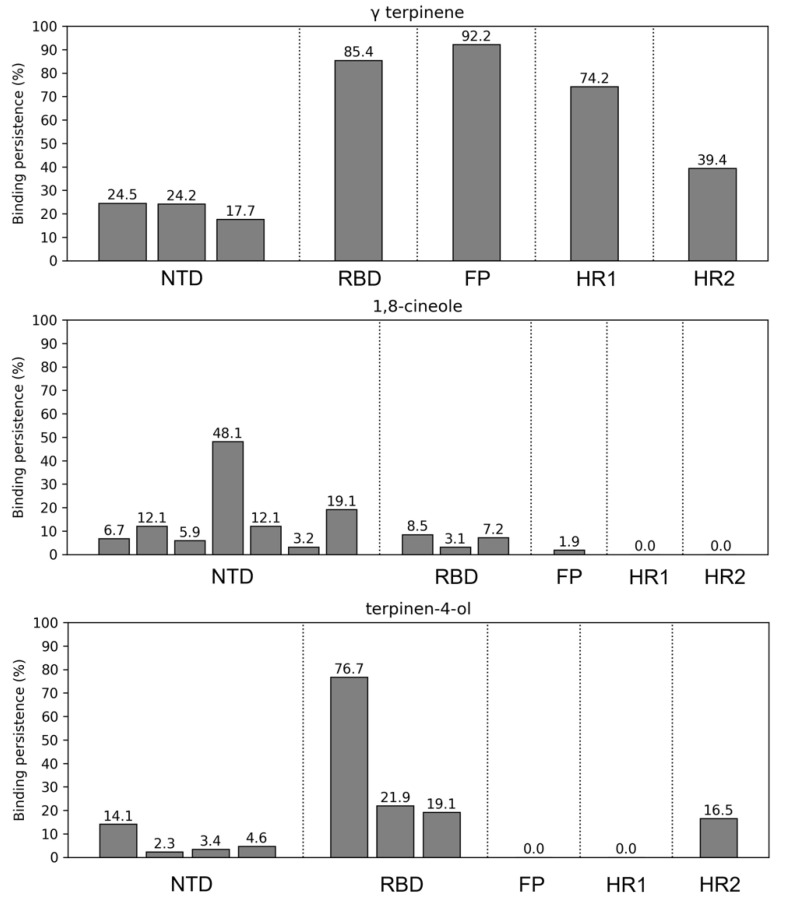
Binding persistence of all TTO molecules contacting the S functional domains during the simulation time. The number of bars represents, for each domain, the number of γ-terpinene, 1,8-cineole, or terpinen-4-ol molecules bound to that domain. The height of the bars indicates the percentage of simulation time in which the molecule was in contact with the domain. Percentage values are also reported on top of bars. (NTD: N-terminal domain, RBD: receptor binding domain, FP: fusion peptide, HR1: heptad-repeats 1, and HR2: heptad-repeats 2).

**Figure 4:**
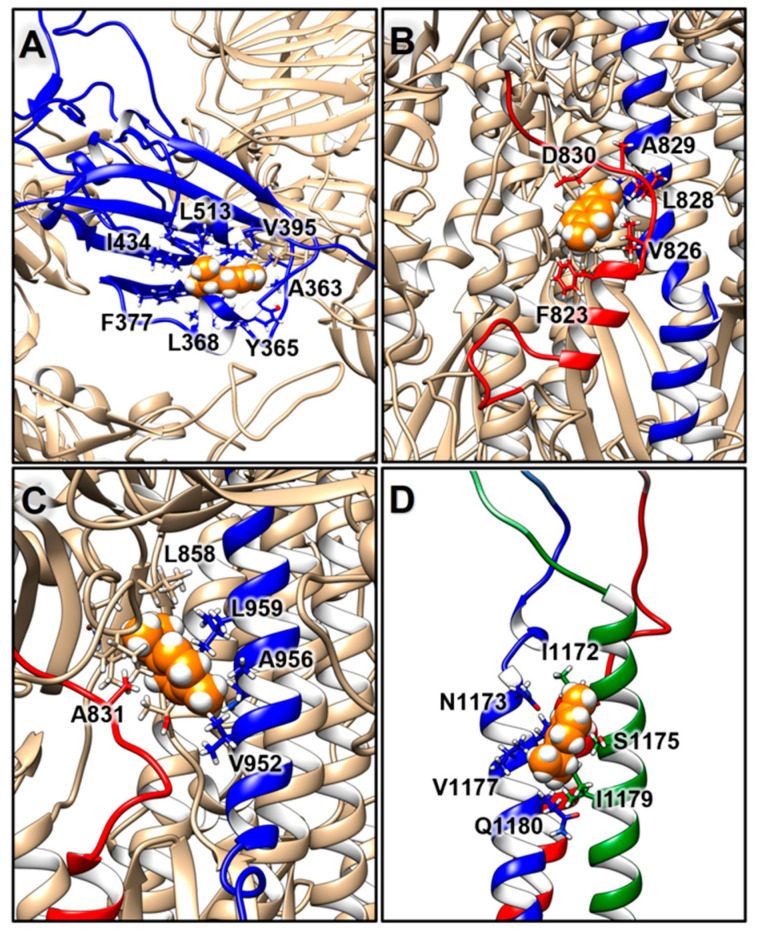
Binding locations observed for γ-terpinene, shown as spheres in orange, on different functional domains of S: (**A**) the RBD, in blue; (**B**,**C**) the S2′/FP and HR1 regions, in red and blue, respectively; (**D**) the HR2 helix bundle, in blue, red, and green. Residues contacted by the ligands are shown as sticks.

**Figure 5:**
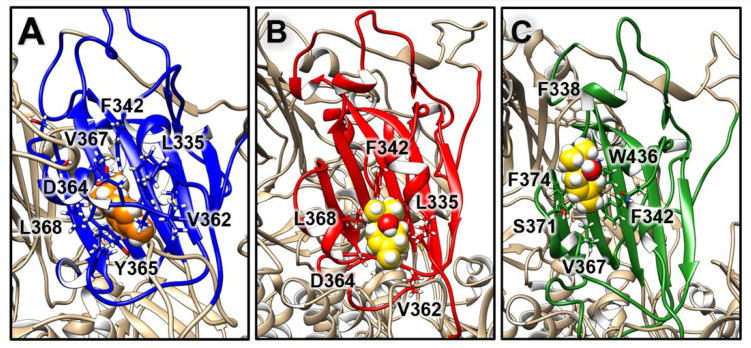
Comparison of the binding poses observed for (**A**) γ-terpinene, colored in orange, and (**B**,**C**) terpinen-4-ol, colored in yellow, on the three S RBDs, highlighted in blue, red, and green.

Further analyses involved the evaluation of the compounds’ interactions with the SARS-CoV-2 S and their influence on the structural dynamics of this protein. An interaction analysis showed that γ-terpinene is the compound that more persistently binds to the S surface (Figure 3). In particular, one γ-terpinene molecule binds for 85.4% of the simulation time to the RBD region of the S monomer B (residues 319–541), suggesting a high affinity of the compound for this region (Figure 3 and Figure 4A). This binding site corresponds to a known fatty acid (FA) binding pocket (residues 330–470 and 500–515) of S, located at each RBD of the trimer [46]. The binding of linoleic acid to these regions was shown to reduce S interactions with ACE2 in vitro, inducing the formation of a more rigid S1 trimer [46,47]. We also observed the binding of two terpinen-4-ol molecules at the interface of the FA binding pockets of the other two RBDs (Figure 3 and Figure 5B,C), which was expected considering the structural similarity between γ-terpinene and terpinen-4-ol. The binding site that was detected suggests that these compounds could interfere to some extent with RBD transition to the open conformation, decreasing the likelihood of S interactions with ACE2. The more hydrophobic character of γ-terpinene, compared to terpinen-4-ol, should favor its insertion within the mostly hydrophobic FA pocket.

One γ-terpinene also binds to a region spanning between the S2′ cleavage site and the fusion peptide (FP) region (residues 808–837), for about 92.2% of the simulation time, and also contacts a segment of the HR1 region (residues 920–970) for 74.2% of the simulation time (Figure 3 and Figure 4B,C). The availability of the S2′ cleavage site for protease priming is crucial for triggering S conformational changes since its cleavage leads to the exposure of the FP’s regions and their insertion in the cell membrane [2]. The attachment of γ-terpinene to this region could thus sterically interfere with S2-protease interactions, causing an early interruption of the subsequent chain of events. Moreover, the small size of this molecule allowed for its insertion within the S surface, reaching the FP region, suggesting that γ-terpinene molecules could also easily find an entry path to the central helical region or internal cavity of the S protein. In particular, the presence of multiple compounds within the inner pocket was suggested as another possible strategy for interference with S conformational changes [48], as already demonstrated for the respiratory syncytial virus fusion (F) protein [49].

For 39.4% of simulation time, γ-terpinene also binds to the HR2 helix bundle (residue 1163 to 1202) (Figure 3 and Figure 4D), although it detaches before the end of the simulation.

On the other hand, while several 1,8-cineole molecules contact the S surface, mostly binding to its NTDs, they only establish transient interactions persisting from about 3.2 to 48.1% of the simulation time (Figure 3). Likewise, apart from contacting the RBDs as described, terpinen-4-ol molecules also transiently contact the S NTD and HR2 regions (Figure 3).

To obtain a general view of the molecules’ distribution around the S protein during the simulation, radial distribution functions (RDFs) of γ-terpinene, 1,8-cineole, and terpinen-4-ol were calculated and averaged for the entire trajectory (Figure S1). This analysis describes how molecules’ densities change as a function of distance from the S surface, and clearly highlights how γ-terpinene molecules are strongly attracted toward the protein, arranging close to the structure, and forming a sharp density peak around 0.8–1.0 nm from the protein (Figure S1). On the other hand, 1,8-cineole and terpinen-4-ol molecules are less gathered around the S surface, with molecules distributing up to 10.0 nm away from the protein (Figure S1). These observations confirm the results shown in Figure 3 and suggest that 1,8-cineole and terpinen-4-ol molecules mainly fluctuate in the solvent without achieving specific interactions on the protein surface.

#### 2.3.3. Influence of TTO on S Mobility

To evaluate if the interaction with TTO compounds and the alterations in membrane thickness influenced S protein structural dynamics, we analyzed the protein motions through a principal component analysis (PCA) [50]. Covariance matrices, generated for both systems using the GROMACS 2020 program [51] and a custom Python script, showed that the presence of TTO compounds can change the protein’s correlation pattern (Figure S2). The S1 subunit of S, including the NTD domains (residues 1–303) and the RBD domains (residues 319–541) of all three monomers, shows an altered pattern of correlated motions in the presence of TTO. In particular, focusing on the RBD domains, the analysis shows a general increase in negatively correlated motions and a decrease in positively correlated motions. Different patterns of positively correlated motions can also be observed in the two systems between the S2 subunits of the three monomers. Positively correlated motions between the S CTDs (residues 1200–1273) and the RBDs present different patterns in the two conditions, and in all three S monomers, there is an increase in the strength of negatively correlated motions between the CTDs and different regions of the S2 subunit (residues 700–1200), including the HR1 and HR2 domains. The observed behavior suggests that the increase in membrane thickness influences S structural dynamics. This evidence is also supported by the RMSF analysis, indicating that the S TM and CP domains, in direct contact with the membrane, exhibit higher flexibility in the presence of TTO (Figure S3).

To better understand the differences observed in the dynamic correlations of the protein, salt bridge networks stabilizing the S domains have been analyzed (Figure S4). Salt bridges usually contribute to constraining motion and protein flexibility; therefore, the presence or absence of these electrostatic interactions can structurally influence the protein conformation, subsequently affecting the overall protein function [52]. The S NTDs, RBDs, and CTDs show the appearance and disappearance of several salt bridges in the presence of TTO, and most of the salt bridges which are conserved in both simulations show different percentages of persistence (Figure S4). However, 5 highly stable salt bridges (Asp290-Arg273, Glu169-Lys129, Asp53-Lys195, Asp398-Arg355, and Asp442-Arg509) were observed in the NTD and RBD of each of the three S monomers. Remarkably, ionic interactions between Asp290-Arg273 and Asp398-Arg355 could contribute to the stability of the RBD in closed conformation, as suggested by a structural analysis performed on several published S structures [53]. On the other hand, the HR2 domain only shows the appearance of two additional salt bridges and minor variations in the salt bridges’ persistence in the presence of TTO, while negligible differences can be observed in the HR1 domain in both conditions (Figure S4).

## 3. Discussion

In the last two years, following the spread of the COVID-19 pandemic, the use of disinfectant agents has become a worldwide daily practice. Enveloped viruses such as SARS-CoV-2 are generally more sensitive than naked viruses to disinfectants since their lipid membrane causes the virus to be more susceptible to chemical and physical conditions [16]. Different disinfectant agents commercially available nowadays have shown efficacy against the Middle East respiratory syndrome coronavirus (MERS-CoV), SARS-CoV-1, and SARS-CoV-2 [16,54,55,56]. The virucidal effect of these substances is mainly based on protein denaturation and disruption of the viral lipid envelope, owing to their amphiphilic properties facilitating their entry into lipid membranes [16,17,54]. The WHO and the FDA recommended the use of alcohol-based sanitizers to contain the spread of SARS-CoV-2 [12,13], but the prolonged and excessive use of these products can cause skin dryness and damage which favors the entry of other harmful microbes [14,15]. Foam or gel formulations reduce the risk of the skin dryness and irritation associated with liquid preparations, but the applied volume and the required drying time deeply affect their efficacy [54]. The possible environmental impacts caused by the increased use of these chemicals are also a matter of concern [14]. On the other hand, many plant-derived compounds possess known antimicrobial properties, making them potentially useful as biodegradable and non-toxic natural disinfectants [17]. In particular, essential oils from different aromatic herbs or plants have shown antiviral activity against coronaviruses, including SARS-CoV-1 [57,58,59], and different reviews have discussed the possible use of essential oils also against SARS-CoV-2 [57,60,61].

Among essential oils, the extract of the *M. alternifolia* tree, commonly called Tea tree oil, possesses great potential as a natural disinfection agent given its well-known antibacterial, antifungal, and antiviral activities [18,22,23,24,25,26,27,28,29,62]. Its lipophilic nature enables penetration into the skin, making it suitable for topical use in treating mucosal and cutaneous infections [18,30,33]. TTO-based handwash formulations have also been evaluated for their possible use in hospital or health care settings. In particular, a handwash study showed that either a product with 5% TTO and 10% alcohol, or a solution of only 5% TTO in water, performed significantly better than soft soap in reducing bacterial proliferation [63]. Another recent randomized trial suggested that the use of a 10% TTO disinfectant resulted in a higher antimicrobial effect than 83% alcohol-based gel disinfectant or a benzalkonium chloride foam disinfectant [64].

In this work, we proposed the use of TTO as a novel natural disinfectant agent against coronaviruses, also suggesting its applicability against SARS-CoV-2. Indeed, the results obtained from our in vitro virucidal assays showed that, at the low concentration of 3.33% and already after 5 min exposure, TTO exhibits strong virucidal effects against FCoVII, with 99.97% of virus inactivation (Table 1). The same TTO concentration also exerted similar effects against HCoV-OC43, with 83.41% inactivation after 5 min, which increased up to 95.83% after 30 min. These effects are comparable to those obtained using a disinfectant with known antimicrobial activity, commonly used in medical settings (Table 1). Lower concentrations of TTO resulted in a reduced efficacy against the two tested coronaviruses (Table 1). Notably, the most effective virucidal concentration of alcohols ranges typically between 60–90% *v*/*v*, while below 50% *v*/*v*, alcoholic solutions are generally considered ineffective against microorganisms [54]. On the contrary, our TTO-EtOH formulation A is extremely virucidal already at the low TTO concentration of 3.33% and with only a low ethanol percentage of 5.33% (Table 1).

Considering the structural similarities between coronaviruses [3], we believed these results could also be extendable to SARS-CoV-2. To evaluate this hypothesis, we applied in silico techniques to explain a possible inhibition mechanism carried out by TTO on the outer region of the SARS-CoV-2 envelope. Antiviral activity of TTO is indeed mainly attributed to the inhibition of viral attachment and fusion to the host cell, involving the impairment of key envelope macromolecules [25,38]. As an example, molecular docking and MD simulations suggested that the TTO-mediated inhibition of the influenza A virus’s (H_1_N_1_) entry into host cells could be due to terpinen-4-ol interactions with the viral haemagglutinin protein [25]. The compound is supposed to bind to a cavity close to the fusion peptide, stabilizing the protein in the prefusion conformation and hindering the conformational rearrangements needed for viral entry [25].

In this paper, to describe TTO interactions with the outer regions of SARS-CoV-2, we performed GaMD simulations [44] of a SARS-CoV-2 S model inserted in a viral membrane [45], randomly distributing around the protein several molecules of three main components of TTO, γ-terpinene, terpinen-4-ol, and 1,8-cineole (Figure 1). These three components were also experimentally tested against FCoVII and HCoV-OC43, confirming that at the highest concentration tested (3.33%) they all possess a time-dependent virucidal activity against these two coronaviruses, causing a 90–99% virus inactivation (Table S2).

The simulation results confirmed that the three compounds easily enter the viral membrane and alter its thickness profile (Figure 2). Their insertion could lead to a modification in the molecular organization of the viral envelope and influence the efficiency of the viral–cell membrane fusion process. Indeed, the alteration of membranes is an antimicrobial mechanism already hypothesized for TTO, since its lipophilic terpene components tend to partition into bilayers disrupting their typical structure and function [36].

Interaction analysis and RDFs calculations showed that γ-terpinene molecules are more attracted toward the S surface than terpinen-4-ol or 1,8-cineole (Figure 3 and Figure S1). Several γ-terpinene molecules were persistently bound to the S protein during the simulation (Figure 3 and Figure 4), influencing the protein’s structural correlation patterns and salt bridge networks (Figures S2 and S4). γ-terpinene specifically targeted four key structural regions of S: the RBD domain, the S2′/FP cleavage protease site, the HR1 helical region, and the HR2 helix bundle (Figure 4). Since these domains are involved in the process of viral and cell membrane fusion, we hypothesize that γ-terpinene may act at different levels, altering the sequence of events leading to the entry of the virus (receptor recognition, S cleavage and activation, fusion peptide insertion, and HR1 and HR2 assembly into the pore-inducing 6-HB) [2]. This observation is in line with experimental results obtained on the enveloped virus HSV-1, indicating that γ-terpinene induces a >96% reduction in plaque formation when pre-incubated with the virus, while terpinen-4-ol and 1,8-cineole showed a lower efficacy [38].

Notably, the binding site achieved by γ-terpinene on one S RBD corresponds to a known fatty acid binding pocket [46]. The binding of linoleic acid to this region induces a so-called “locked” S conformation, characterized by maximized inter-subunit contacts between the RBDs and a more rigid S1 trimer, reducing S interactions with ACE2 in vitro [46,47].

As opposed to γ-terpinene and terpinen-4-ol, 1,8-cineol did not show particular affinity for the S protein, but it was able to easily enter the viral membrane (Figure 2 and Figure 3). This is in agreement with the mechanism of the TTO-induced inhibition of the methicillin-resistant *S. aureus* (MRSA), in which the essential oil caused a destabilization of the bacterial membrane [18]. The greatest effects were observed when using 1,8-cineole alone, suggesting that the antimicrobial activity of this molecule may be specifically directed towards membranes, allowing for their destabilization and permeabilization, and facilitating the entry of other active components.

Due to the cytotoxic effects of TTO and its components on CRFK cells, the mixture of each tested product and FCoVII was diluted before infecting the cells. This resulted in a significant reduction of the original titer of FCoVII (6 Log), as shown in the values of the virus controls that were obtained, and could have underestimated the virucidal potency of TTO and its components. However, we demonstrated that all tested products still have significant virucidal activity against FCoVII (% virus inactivation: TTO: ≥99.97; and terpinen-4-ol: ≥99.68) (Table 1 and Table S2).

The absence of a direct experimental confirmation regarding TTO inhibition of SARS-CoV-2 may represent a weakness of this research. However, the potential effects on SARS-CoV-2 may be justifiably inferred from the obtained simulation results, and supported by the assays on the surrogate coronavirus models. Indeed, surrogate coronavirus models for SARS-CoV-2 (e.g., FCoVII and HCoV-OC43) have been commonly exploited during the pandemic to avoid the added costs and biosafety concerns related to SARS-CoV-2 manipulation, which would require biosafety level 3 (BSL-3) conditions [65,66]. In particular, HCoV-OC43 belongs to the same Betacoronavirus genus as MERS-CoV and SARS-CoV-1/-2, and was identified early on in the current pandemic by the American Society for Testing and Materials (ASTM) as a preferred surrogate for SARS-CoV-2 on account of its shared characteristics. In fact, similar to SARS-CoV-2, HCoV-OC43 is transmitted via respiratory aerosols and droplets, replicates in the human respiratory epithelium, and is relatively resistant to disinfectants [67,68,69]. Although FCoVII belongs to a different genus (i.e., Alphacoronavirus) and has a different tropism than SARS-CoV-2, both viruses are closely related by key genome characteristics and receptor usage, and their Spike protein RBDs show similar furin cleavage sites [43].

Certainly, simulation methods bear known intrinsic limits, as they atomistically describe only a small portion of the system, and thus do not include all the molecular components of the virus. Furthermore, all-atoms models only allow for the simulation of very short timescales compared to experiments due to the high computational cost of calculations. Nonetheless, current MD sampling strategies and force fields allowed in silico predictions to gain reliability in characterizing biomolecular interactions and binding processes [70].

In conclusion, in this study, we experimentally demonstrated for the first time the strong in vitro virucidal activity of a low concentration of TTO and its individual components against two coronaviruses. Moreover, considering the results obtained through the GaMD simulations, we hypothesize that its efficacy could be easily transferred to SARS-CoV-2, suggesting that the TTO could be efficiently used as a safe disinfectant agent that limits the diffusion of enveloped pathogens.

## 4. Materials and Methods

### 4.1. Tea Tree Oil Formulations, Cells and Viruses

Different TTO-EtOH formulations (A–C, Table 1), characterized by scalar TTO and EtOH concentrations, were used to carry out the in vitro virucidal assays. TTO dilutions (3.33%, 0.66%, and 0.13%) were obtained starting from pure TTO (Sigma-Aldrich W390208, Saint Louis, MO, USA), while that of EtOH (5.33%, 1.06%, and 0.21%) were produced starting from 80% of EtOH in distilled water (Merck K35016883, Rahway, NJ, USA). Product specifications for TTO can be found at https://www.sigmaaldrich.com/specification-sheets/408/492/W390208-BULK-K____ALDRICH__.pdf (accessed on 30 May 2022) and https://www.sigmaaldrich.com/deepweb/assets/sigmaaldrich/quality/spectra/141/655/ATIR0018505.pdf (accessed on 30 May 2022). γ-terpinene (Sigma-Aldrich 223190), 1,8-cineole (Sigma-Aldrich PHR1828) and terpinen-4-ol (Sigma-Aldrich W224820) were diluted as described for pure TTO at 3.33% and 0.66%. The disinfectant ISACLEAN^®^ (Cantel Medical S.R.L., Pomezia, Italy), a product with proven virucidal effects, was used as a reference agent (composition: isazone, benzalkonium chloride 50%, chlorhexidine gluconate, and isopropanol). 

Vero E6 (African green monkey kidney cells; CRL-1586™. ATCC, 10801 University Boulevard, Manassas, VA, USA) and CRFK (Crandell–Rees feline kidney cells; Kind gift from Prof. Buonavoglia, University of Bari) were cultured in Dulbecco’s modified Eagle’s medium (DMEM) supplemented with 10% fetal bovine serum (FBS), antibiotic–antimycotic mixture, HEPES buffer, l-glutamine, and gentamicin solution. The cells were incubated at 37 °C in a humidified atmosphere of 5% CO_2_. Feline coronavirus (FCoVII), a kind gift from Prof. Buonavoglia (University of Bari), was propagated in CRFK cells and harvested after the appearance of cytopathic effects (CPE) at 48 hpi. FCoVII titer was determined using the Reed and Muench method [71] and expressed as 50% tissue culture infective dose (TCID_50_/mL). HCoV-OC43 was a kind gift from Prof. Baldanti (S. Matteo Hospital, Pavia) and was propagated in Vero E6. Since HCoV-OC43 rarely shows an obvious CPE in infected cell lines [66,72], the virus was harvested at 4 days post-infection according to previous study [73]. All viral stocks were stored at −80 °C. Both FCoVII and HCoV-OC43 can be handled at BSL-2 and provide an alternative to highly pathogenic coronaviruses for the screening of antivirals and/or analysis of virucidal effects of disinfectants agents.

### 4.2. In Vitro Virucidal Assays

Two different coronaviruses, FCoVII and HCoV-OC43, were used to examine the virucidal effects of different TTO-EtOH formulations (A–C), EtOH (formulation D), a reference disinfectant (Table 1), and individual TTO components (Table S2). The virucidal activity assays were performed in three intervals (5, 15, and 30 min) of exposure and contact of the viruses with the different TTO-EtOH formulations (A–C) and formulations of individual compounds, with fixed amounts of FCoVII (6 Log TCID_50_/mL) and HCoV-OC43 (5 Log RNA copies/mL). An amount of 100 µL of each virus was mixed with 100 μL of each formulation. Virus controls with 100 µL medium in place of TTO-EtOH were included. Determination of virucidal effects of EtOH was also carried out by mixing 100 µL of each virus with an equal volume of EtOH at a concentration of 5.33% (formulation D) for 30 min. At the end of the exposure times, the virucidal activity was stopped by addition of 900 μL of ice-cold cell culture medium and later determined by means of end point dilution titration in microtiter plates (FCoVII/ CRFK cells) or RT/Real Time PCR (HCoV-OC43/ Vero E6 cells). In particular, the medium was removed from 96-well culture plates previously seeded and incubated for 24 h at 37 °C in 5% CO2 with CRFK or Vero E6 cells and replaced with three-fold serial dilutions of FCoVII- or HcoV-OC43-TTO mixtures in triplicate, starting from a non-cytotoxic concentration (formulation A and D) and 1 dilution factor (formulations B and C) on a logarithmic scale at base 10. Each plate included uninfected control cells as an indicator of cell viability. Viral titer reduction of both coronaviruses was presented as the difference between the virus titers after the exposure time with TTO and the control virus titer (medium). Titer of FCoVII was measured after CPE appearance at 48 hpi, using the Reed and Muench method [71] and expressed as TCID_50_/mL. Since HCoV-OC43 does not produce evident CPE in Vero E6 cells, and thus the TCID_50_ assay by CPE observation is not applicable for titration, viral titer was measured using RT/Real Time PCR after 96 hpi.

### 4.3. Real-Time, Quantitative Reverse Transcriptase PCR Assay for Absolute Quantitation of Human Coronaviruses OC43

RNA was extracted from Vero E6 cells using Total RNA Purification Kit (NorgenBiotek Corp, Thorold, ON, Canada) and reverse transcribed by high-Capacity cDNA Reverse Transcription Kit (Applied Biosystems, Waltham, MA, USA). Type-specific primers and a probe for N gene HCoV-OC43 were added to the Probe PCR Master Mix (Roche, Basel, Switzerland) at 500 and 250 nM, respectively, in a final volume of 20 μL. The primers and probe sequences used for N gene of HCoV-OC43 were the following:Forward5′-AGCAGACCTTCCTGAGCCTTCAAT-3′;Reverse5′-AGCAACCAGGCTGATGTCAATACC-3′;Probe5′-/56-FAM/TGACATTGTCGATCGGGACCCAAGTA/36-TAMSp/-3′.

The standards for HCoV-OC43 were obtained by cloning the 600 bp of viral N gene into the pCR2.1 plasmid using a TOPO TA cloning kit (Invitrogen Corporation, San Diego, CA, USA). A linear distribution (r = 0.99) was obtained between 10 and 10^8^ copies of HCoV-OC43-DNA. Viral titer values were Log transformed for analysis and data was expressed as the Log number of HCoV-OC43 copies per mL.

### 4.4. GaMD Simulations of the Spike Glycoprotein in a Viral Membrane

GaMD simulations were performed using a complete model of the SARS-CoV-2 S glycoprotein in prefusion conformation, with RBDs in closed configuration and including glycosylation and palmitoylation sites [45]. The protein sequence belongs to the original wild-type strain [1]. The GaMD method allows unconstrained enhanced sampling of biomolecular systems and has been successfully applied to outline ligand binding pathways during simulations [44,74]. The method works by adaptively adding a harmonic boost potential that smooths the system potential energy surface, which reduces energy barriers and accelerates simulations by orders of magnitude, without the need to set predefined reaction coordinates.

Topology and coordinate files for the protein, inserted in a 230 × 230 Å membrane, were generated using the Membrane Builder tool of the CHARMM-GUI (https://www.charmm-gui.org/ (accessed on 30 May 2022); Lehigh University, Bethlehem, Palestine) interface [75]. The CHARMM36m force field for proteins [76], lipids [77], and carbohydrates [78] was used to parametrize the protein-membrane system. Membrane composition mimics that of a viral envelope, including: cholesterol (30%), 3-palmitoyl-2-oleoyl-d-glycero-1-phosphatidylcholine (6%), 2,3 dipalmitoyl-d-glycero-1-phosphatidylcholine (4%), 3-palmitoyl-2-oleoyl-d-glycero-1-phosphatidylethanolamine (18%), 2,3 dipalmitoyl-d-glycero-1-phosphatidylethanolamine (12%), 3-palmitoyl-2-oleoyl-d-glycero-1-phosphatidylserine (6%), 2,3 dipalmitoyl-d-glycero-1-phosphatidylserine (4%), and sphingomyelin d18;1/16;0 (20%) [45].

The protein–membrane system was inserted in a rectangular box filled with TIP3P water molecules [79] and neutralized with 0.15 M of NaCl ions. The final system included 2,087,887 atoms. To remove unfavorable interactions, the system was minimized in ten runs, each including 2000 steps. A constraint of 20.0 kcal/mol was initially applied on each atom, sequentially halved in the subsequent runs and finally removed in the last one. The minimized system has been thermalized in a canonical ensemble (NVT) using a timestep of 1.0 fs, gradually increasing the temperature from 0 to 310 K every 30 ps using Langevin dynamics [80] and applying a constraint of 5.0 kcal/mol on protein and membrane atoms. The system was then equilibrated in an anisotropic NPT (NPT-A) ensemble using the Nosè–Hoover Langevin piston method [81,82] and a constant pressure of 1.0 atm, gradually releasing the constraints applied on the protein and membrane every 250.0 ps during a 2250.0 ps run. Then, the timestep was increased to 2.0 fs and the system was simulated for 10.0 ns using classical MD before starting 150 ns of dual-boost Gaussian accelerated Molecular Dynamics (GaMD) [44]. GaMD simulation included 2.0 ns of classical MD preparation, 50.0 ns of GaMD equilibration, and 100.0 ns of GaMD production. The upper and lower limits for the standard deviation of the total boost potential were maintained to the default value of 6.0 kcal/mol. Electrostatic interactions were calculated using the PME method [83], while the cut-off for non-bonded interactions was set to 12.0 Å. At the end of the simulation, a representative frame of the protein–membrane system was extracted from the production phase of the simulation using the cluster module of the GROMACS 2020 MD package [51] and the gromos method [84]. This equilibrated structure was used as a starting point for two other simulations, performed in the absence or presence of several molecules of three representative compounds of TTO (γ-terpinene, terpinen-4-ol, and 1,8-cineole), randomly inserted around the S glycoprotein and the membrane using the Packmol program v.20.010 (http://leandro.iqm.unicamp.br/m3g/packmol/home.shtml (accessed on 2 February 2021); University of Campinas and University of São Paulo, Brazil) [85]. The compound’s structures were retrieved from the PubChem database [86] (Table S1) and their parameters were generated using the CGenFF program (https://cgenff.umaryland.edu) (accessed on 2 February 2021) and the CHARMM general force field [87]. Topologies and coordinates files were generated using the VMD program [88], solvating the systems in rectangular boxes of TIP3P water molecules [79] and neutralizing them with 0.15 M of NaCl ions. The same minimization and equilibration protocol was applied, except that a shorter NPT-A equilibration of 1500.0 ps was performed prior to the 10.0 ns of classical MD. For both systems, 150.0 ns of dual-boost GaMD simulations [44] were carried out as previously described. 

All simulations were performed using the NAMD v. 2.13 program (https://www.ks.uiuc.edu/Research/namd/ (accessed on 2 February 2021); University of Illinois at Urbana–Champaign, IL, US) [89] on the ENEA CRESCO6 HPC cluster [90], saving systems coordinates every 1000 steps.

### 4.5. Trajectories Analysis

Principal component analysis (PCA) [50] was performed on the Cα atoms of S using the GROMACS 2020 MD package [51]. Correlation plots were generated using a custom Python script. Salt bridges were analyzed using the VMD v. 1.9.3 Salt Bridges Tool [88]. Membrane thickness was evaluated using the VMD MEMBPLUGIN Tool (https://www.ks.uiuc.edu/Research/vmd/ (accessed on 2 February 2021); University of Illinois at Urbana, Champaign, IL, USA) [91]. Binding persistence of TTO molecules with each S domain was analyzed using a custom Tcl script in VMD, setting the distance threshold for identifying a contact to 4.0 Å. Radial distribution functions (RDFs) for the three TTO molecules were calculated using the GROMACS 2020 MD package (https://www.gromacs.org (accessed on 2 February 2021); University of Groningen, The Netherlands) [51], setting Cα atoms of S as the reference set of positions. Plots were generated using custom Python scripts and images were created using the software VMD [88] or Chimera v. 1.14 (https://www.cgl.ucsf.edu/chimera/ (accessed on 2 February 2021); University of California, San Francisco, CA, USA) [92].

## Figures and Tables

**Table 1:** Virucidal activity of TTO against FCoVII and HCoV-OC43.

Formulation (TTO%, EtOH%)	Time of Exposure (min)	FCoVII (Log TCID_50_/mL)				HCoV-OC43 (Log RNA Copies/mL)			
		Virus Exposed to TTO	Virus Control	RV	Virus Inactivation (%)	Virus Exposed to TTO	Virus Control	RV	Virus Inactivation (%)
A * (3.33, 5.33)	5	<0.50	4.00	≥3.50	≥99.97 *	2.58	3.36	0.78	83.41
	15	<0.50	4.00	≥3.50	≥99.97 *	2.56	3.28	0.72	80.96
	30	<0.50	4.00	≥3.50	≥99.97 *	2.78	4.16	1.38	95.83
B (0.66, 1.06)	5	3.75	3.50	0.00	0.00	3.79	3.36	0.00	0.00
	15	3.75	4.00	0.25	43.76	3.69	3.28	0.00	0.00
	30	4.00	4.50	0.50	68.36	4.78	5.13	0.35	55.36
C (0.13, 0.21)	5	3.50	3.50	0.00	0.00	3.24	3.36	0.00	0.00
	15	4.00	4.00	0.00	0.00	3.45	3.28	0.00	0.00
	30	4.50	4.50	0.00	0.00	4.80	5.13	0.33	53.28
D (0.00, 5.33)	5	ND	ND	ND	ND	ND	ND	ND	ND
	15	ND	ND	ND	ND	ND	ND	ND	ND
	30	4.00	4.00	0.00	0.00	4.12	4.05	0.00	0.00
Disinfectant **	5	<0.50	3.50	≥3.00	≥99.90 *	1.90	4.03	2.13	99.26

Reduction value (RV) is the reduction of the virus in test product (formulations A–D) compared to the virus control not exposed to the test product. The virus percentage inactivation is calculated using the formula 1 − (1/10^RV^) × 100%. * Due to cytotoxicity of the tested formulation A, the detection limit did not allow for the detection of higher FCoVII reduction. ** Disinfectant composition: isazone, benzalkonium chloride 50%, chlorhexidine gluconate, and isopropanol. ND = not determined.

## Data Availability

Not applicable.

## Supplementary Materials

The following supporting information can be downloaded at: https://www.mdpi.com/article/10.3390/molecules27123786/s1, Table S1: Structural information for the three simulated TTO molecules; Table S2: Virucidal activity of single components of TTO against FCoVII and HCoV-OC43; Figure S1: Radial distribution functions of the three TTO molecules; Figure S2: Correlation matrices calculated for the S protein; Figure S3: RMSF analysis for the three Spike monomers; Figure S4: Salt bridges analysis for the main S domains.
