# Supplementary material for: Potential Use of Tea Tree Oil as a Disinfectant Agent against Coronaviruses: A Combined Experimental and Simulation Study
Source: Molecules. 2022 Jun 12;27(12):3786. doi: 10.3390/molecules27123786 (PMC9228983; doi:10.3390/molecules27123786)
Supplement: Supplementary file 1 [file molecules-27-03786-s001.zip › molecules-1771555-supplementary.pdf]

molecules

Supplementary Material

Potential Use of Tea Tree Oil as a Disinfectant Agent Against Coronaviruses: a Combined Experimental and Simulation Study

Alice Romeo [1], Federico Iacovelli [1], Carolina Scagnolari [2,3], Mirko Scordio [2,3], Federica Frasca [2,3], Roberta Condò [4], Serena Ammendola [1], Roberta Gaziano [5], Maurizio Anselmi [6], Maurizio Divizia [6] and Mattia Falconi [1,*]

[1] Department of Biology, University of Tor Vergata, 00133 Rome, Italy; alice.romeo@uniroma2.it (A.R.); federico.iacovelli@uniroma2.it (F.I.); serena.ammendola@uniroma2.it (S.A.)
[2] Laboratory of Virology, Department of Molecular Medicine, Sapienza University of Rome, 00185 Rome, Italy; carolina.scagnolari@uniroma1.it (C.S.); mirko.scordio@uniroma1.it (M.S.); federica.frasca@uniroma1.it (F.F.)
[3] Istituto Pasteur Italia, 00161 Rome, Italy
[4] Department of Clinical Sciences and Translations Medicine, University of Tor Vergata, 00133 Rome, Italy; roberta.condo@uniroma2.it
[5] Department of Experimental Medicine, University of Tor Vergata, 00133 Rome, Italy; roberta.gaziano@uniroma2.it
[6] Department of Biomedicine and Prevention, University of Tor Vergata, 00133 Rome, Italy; maurizio.anselmi@uniroma2.it (M.A.); divizia@uniroma2.it (M.D.)
* Correspondence: falconi@uniroma2.it; Tel.: +39-06-7259-4025

Copyright: © 2022 by the authors. Licensee MDPI, Basel, Switzerland. This article is an open access article distributed under the terms and conditions of the Creative Commons Attribution (CC BY) license (https://creativecommons.org/licenses/by/4.0/).

Table S1. Structural information for the three simulated TTO molecules.

PubChem CID	Name	SMILES	2D structure
7461	γ-terpinene	CC1=CCC(=CC1)C(C)C	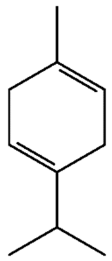

11230	terpinen-4-ol	CC1=CCC(CC1)(C(C)C)O	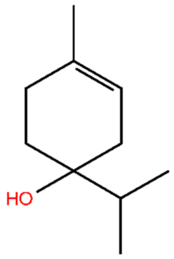
2758	1,8-cineole	CC1(C2CCC(O1)(CC2)C)C	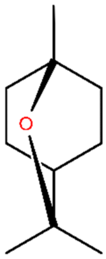

Table S2. Virucidal activity of single components of TTO against FCoVII and HCoV-OC43.

		FCoVII (Log $TCID_{50}$/ml)				HCoV-OC43 (Log RNA copies/ml)			
**TTO Components, %**	**Time of Exposure (min)**	**Virus Exposed to Compound**	**Virus Control**	**RV**	**Virus Inactivation (%)**	**Virus Exposed to Compound**	**Virus Control**	**RV**	**Virus Inactivation (%)**
γ-terpinene, 3.33	5	2.50	3.00	0.50	68.38	3.91	4.39	0.48	66.88
	15	2.00	3.00	1.00	90.00	3.50	4.39	0.89	87.11
	30	2.00	3.00	1.00	90.00	3.35	4.39	1.04	90.87
γ-terpinene, 0.66	5	2.50	3.00	0.50	68.38	3.83	4.39	0.56	72.46
	15	2.50	3.00	0.50	68.38	3.57	4.39	0.82	84.86
	30	2.50	3.00	0.50	68.38	3.30	4.39	1.09	91.87
1,8-cineole, 3.33	5	2.50	3.00	0.50	68.38	3.34	4.39	1.05	91.09
	15	2.50	3.00	0.50	68.38	3.02	4.39	1.37	95.73
	30	1.50	3.00	1.50	96.84	2.90	4.39	1.49	96.76
1,8-cineole, 0.66	5	2.50	3.00	0.50	68.38	3.90	4.39	0.49	67.64
	15	2.50	3.00	0.50	68.38	3.71	4.39	0.68	79.11
	30	2.00	3.00	1.00	90.00	3.55	4.39	0.84	85.54
terpinen-4-ol, 3.33	5	<0.50	3.00	≥2.50	≥99.68*	2.24	4.39	2.15	99.29
	15	<0.50	3.00	≥2.50	≥99.68*	2.23	4.39	2.16	99.30
	30	<0.50	3.00	≥2.50	≥99.68*	1.97	4.39	2.42	99.62
terpinen-4-ol, 0.66	5	3.00	3.00	0.00	0.00	4.01	4.39	0.38	58.31
	15	2.50	3.00	0.50	68.38	3.78	4.39	0.61	75.45
	30	2.00	3.00	1.00	90.00	3.75	4.39	0.64	77.09

All the virucidal experiments were performed mixing an amount of each virus with non-cytotoxic dilution of compounds (1:1). Reduction value (RV) is the reduction of virus in test product compared to virus control not exposed to compound. The virus percentage inactivation is calculated using the formula 1-(1/10LRV) x 100%. Viral titers of FCoVII are expressed as Log TCID50/ml, while viral titers of HCoV-OC43 is indicated as Log RNA copies/ml. * due to cytotoxicity of the tested compound, the detection limit did not allow to detect higher FCoVII reduction.

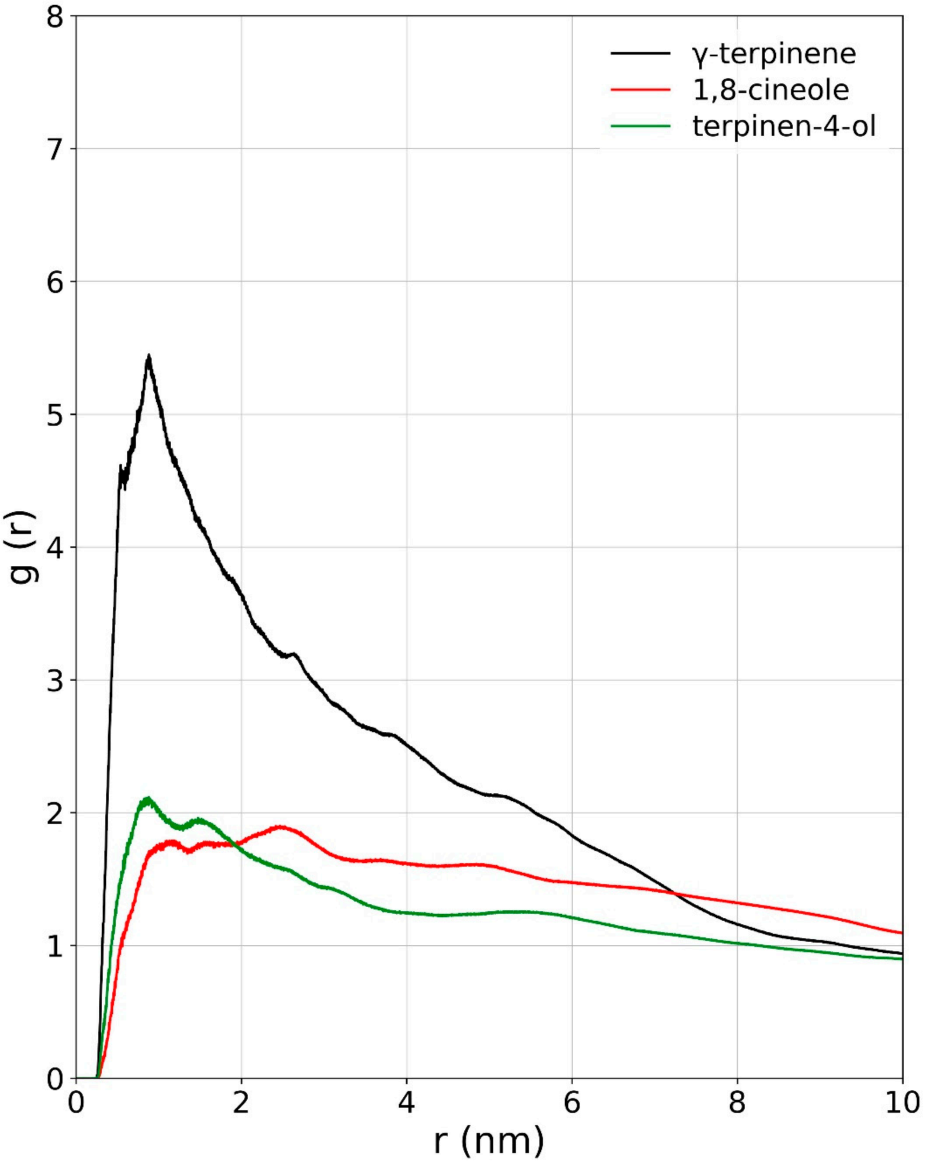

Figure S1. Radial distribution function (RDF) calculated for the three TTO molecules over the S surface during the 150 ns trajectory.

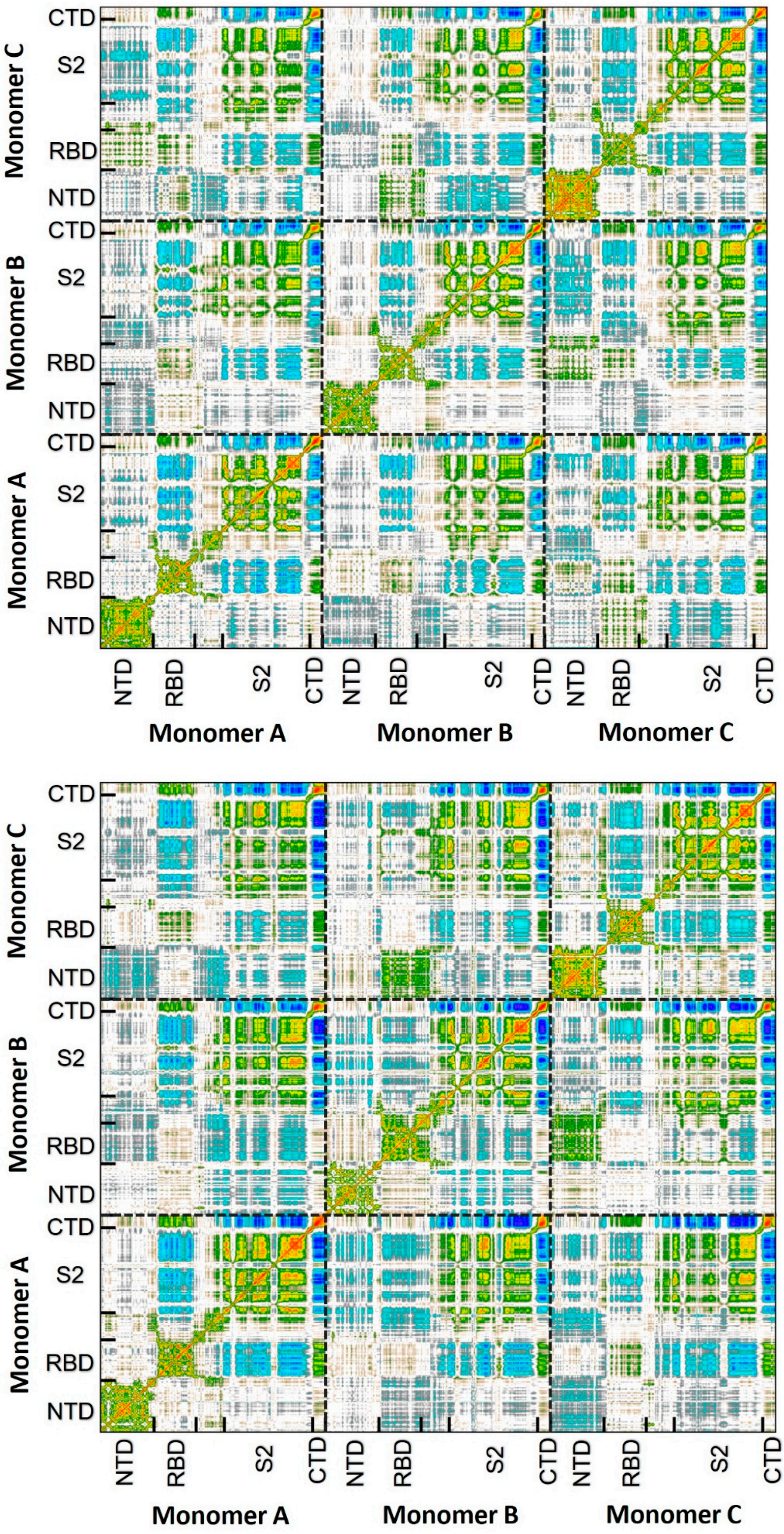

Figure S2. Correlation plots obtained for the S protein in the absence (up) or presence (down) of TTO compounds.

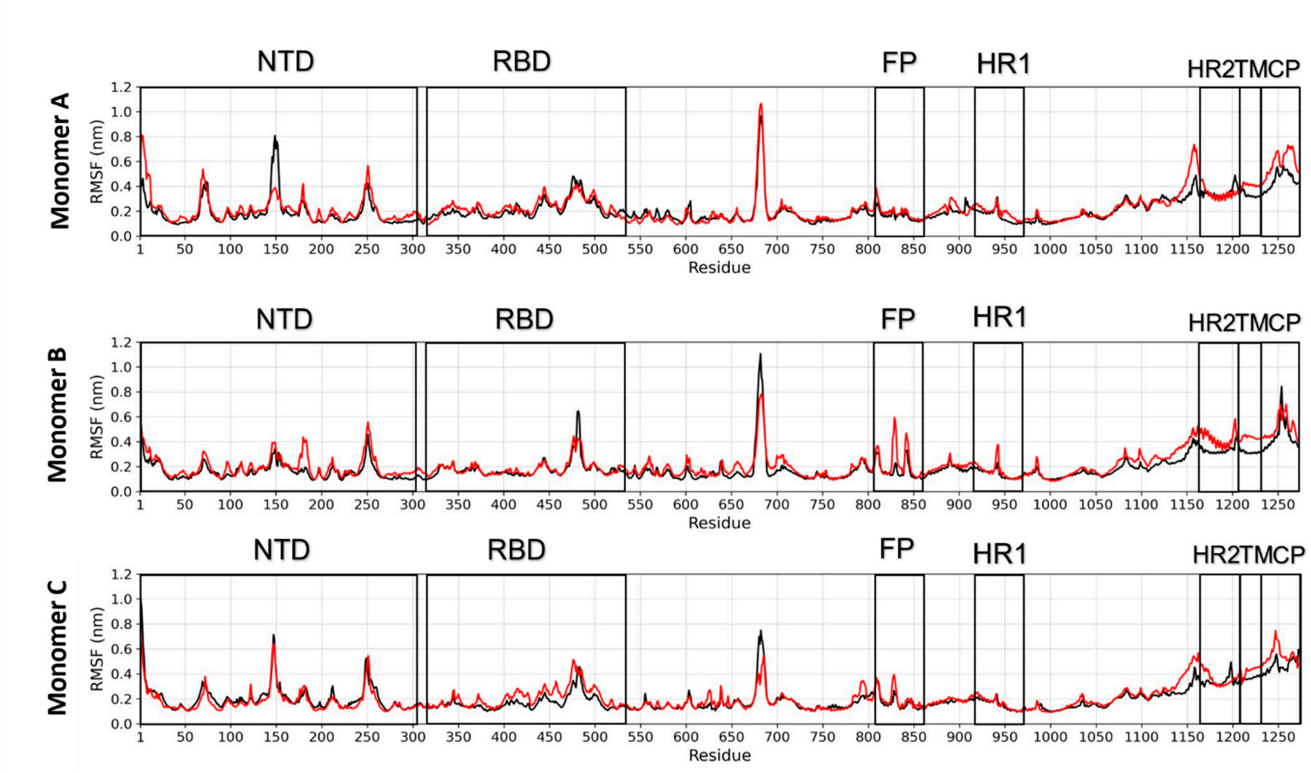

Figure S3. RMSFs calculated for the three S monomers (A, B, C) during the 150 ns GaMD simulations in the absence (black line) or presence (red line) of the TTO molecules. Black squares highlight the different S domains.

Figure S4. Heatmaps representing salt bridges interactions established within each S domain, in the absence or presence of TTO compounds. Heatmaps are colored according to each salt bridges persistence during the simulation time. Black circles indicate differences in the interaction pattern in the two conditions.

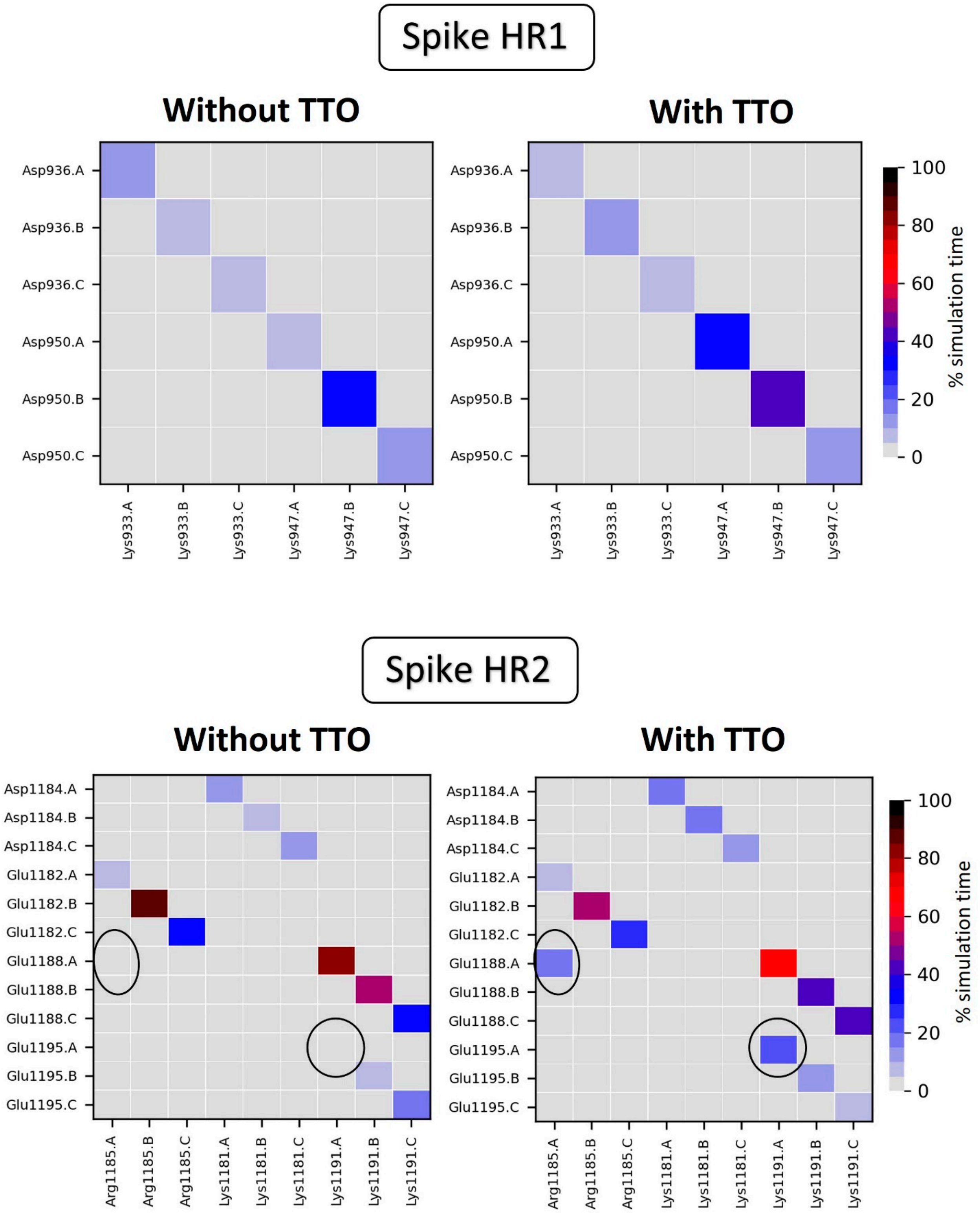

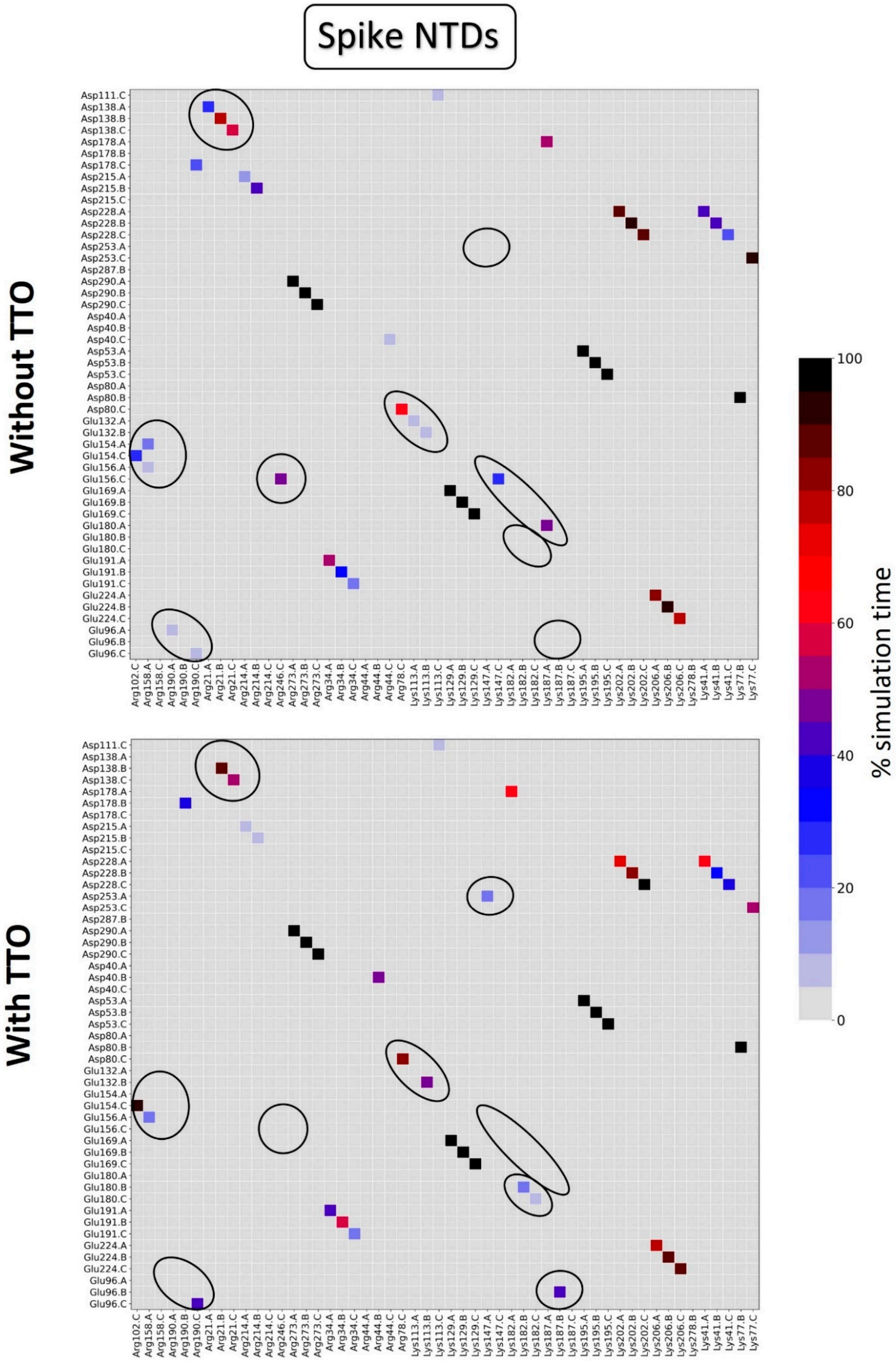
Spike NTDs
Without TTO
With TTO
% simulation time
0
20
40
60
80
100

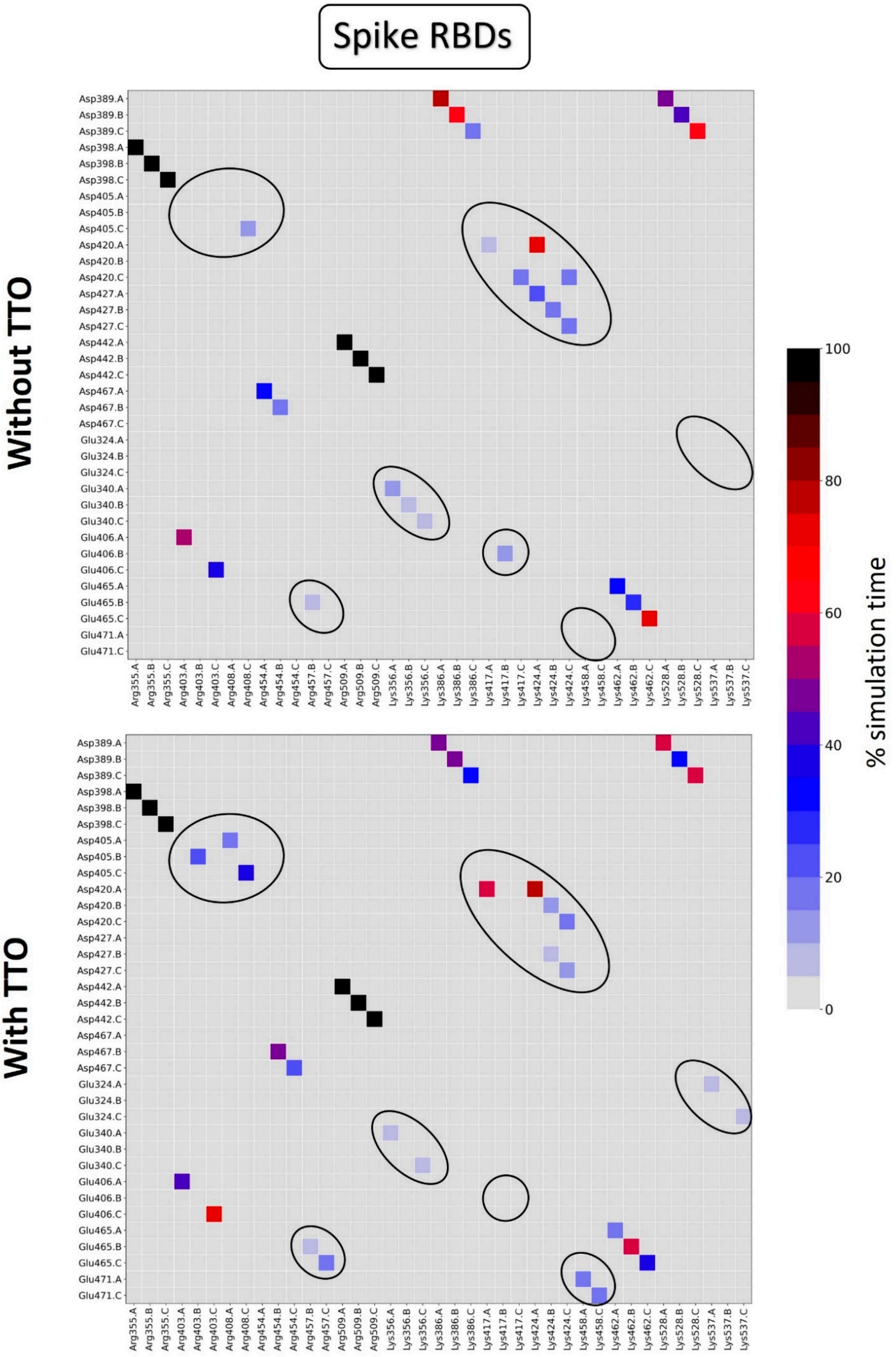
Spike RBDs
Without TTO
With TTO
Asp389.A Asp389.B Asp389.C Asp398.A Asp398.B Asp398.C Asp405.A Asp405.B Asp405.C Asp420.A Asp420.B Asp420.C Asp427.A Asp427.B Asp427.C Asp442.A Asp442.B Asp442.C Asp467.A Asp467.B Asp467.C Glu324.A Glu324.B Glu324.C Glu340.A Glu340.B Glu340.C Glu406.A Glu406.B Glu406.C Glu465.A Glu465.B Glu465.C Glu471.A Glu471.C
Arg355.A Arg355.B Arg355.C Arg403.A Arg403.B Arg403.C Arg408.A Arg408.C Arg454.A Arg454.B Arg454.C Arg457.B Arg457.C Arg509.A Arg509.B Arg509.C Lys356.A Lys356.B Lys356.C Lys386.A Lys386.B Lys386.C Lys417.A Lys417.B Lys417.C Lys424.A Lys424.B Lys424.C Lys458.A Lys458.C Lys462.A Lys462.B Lys462.C Lys528.A Lys528.B Lys528.C Lys537.A Lys537.B Lys537.C
100
80
60
40
20
0
% simulation time

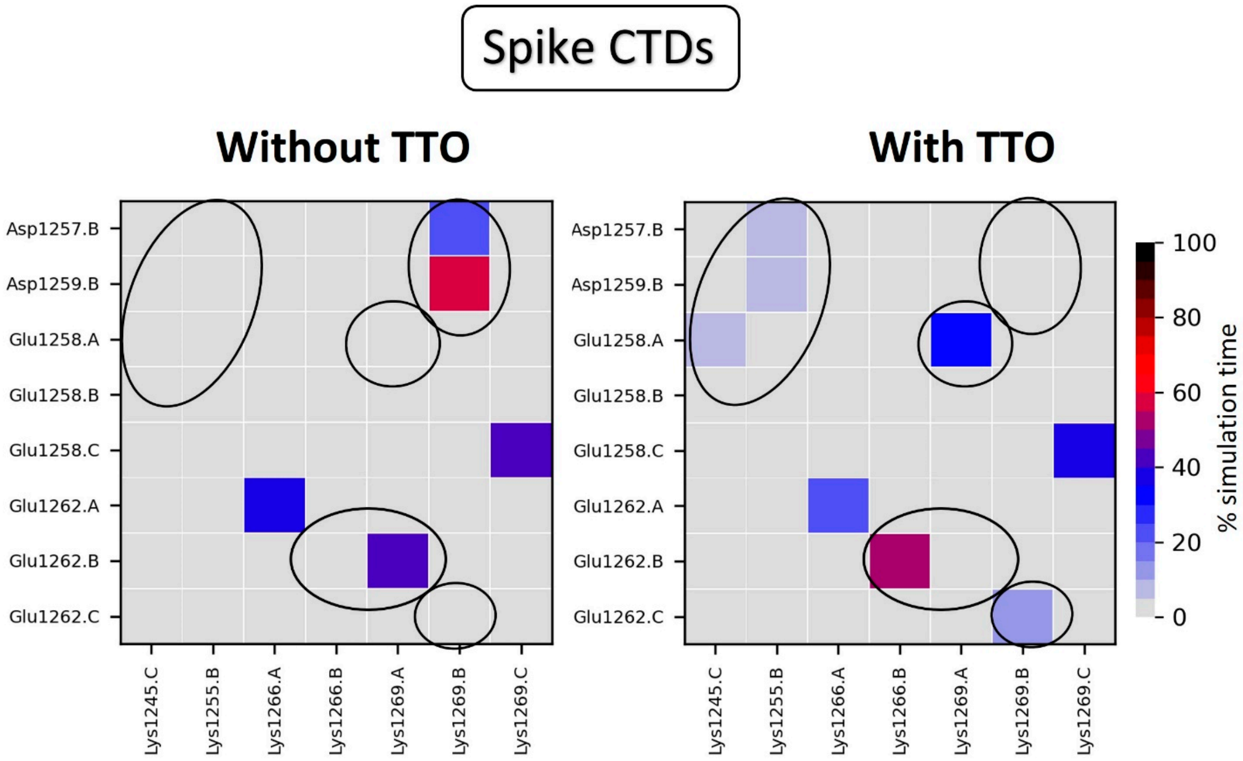

Spike CTDs
Without TTO
With TTO
Asp1257.B
Asp1259.B
Glu1258.A
Glu1258.B
Glu1258.C
Glu1262.A
Glu1262.B
Glu1262.C
Lys1245.C
Lys1255.B
Lys1266.A
Lys1266.B
Lys1269.A
Lys1269.B
Lys1269.C
100
80
60
40
20
0
% simulation time
